# The impact of green finance on total factor carbon emission reduction efficiency in China

**DOI:** 10.1007/s11356-023-30734-y

**Published:** 2023-11-29

**Authors:** Xiaoqiang Hu, Yihan Xia, Jiahui Guo, Yexi Zhong, Jianbo Mao

**Affiliations:** 1 Management Science and Engineering Research Center, Jiangxi Normal University, Nanchang, 330022 Jiangxi China; 2 Jiangxi Institute of Economic Development, Jiangxi Normal University, Nanchang, 330022 Jiangxi China; 3 School of Geography and Environment, Jiangxi Normal University, Nanchang, 330022 Jiangxi China; 4 Chinese Universe Publishing and Media Group Co., Ltd., Nanchang, 330000 Jiangxi China

**Keywords:** Green finance, Total factor carbon emission reduction efficiency, Spatial spillover effect

## Abstract

Based on an analysis of the relationship between green finance and total factor carbon emission reduction efficiency, this article measures the levels of green finance and total factor carbon emission reduction efficiency in 30 provinces and cities in China. It also establishes a spatial Durbin model to quantitatively explore the impact of green finance on China’s total factor carbon emission reduction efficiency. The results indicate that currently, green finance and total factor carbon emission reduction efficiency in China follow a distribution pattern of high in the east, medium in the central region, and low in the west. The impact of green finance on total factor carbon emission reduction efficiency demonstrates a U-shaped relationship, and the spatial spillover effect between the two displays a similar U-shaped trend. The mechanism analysis demonstrates that green finance exerts a U-shaped influence on the efficiency of reducing total factor carbon emissions through the interplay of technological progress and technical efficiency enhancements. Other variables, such as research and development investment, comprehensive energy consumption, human capital, infrastructure construction, and government regulation, also have an impact on total factor carbon emission reduction efficiency. Therefore, it is recommended that regions strengthen their green finance initiatives, support efforts to carbon emission reduction, and contribute towards achieving the “dual-carbon” goal.

**Supplementary Information:**

The online version contains supplementary material available at 10.1007/s11356-023-30734-y.

## Introduction

Since the reform and opening up, China has undergone more than 40 years of economic and social development. This progress has led to substantial improvements in its economic development, propelling China to become the world’s second-largest economy. However, rapid economic growth has also brought severe environmental and climate problems. Being the world’s largest energy consumer and carbon emitter, China’s environment is reaching its capacity due to heavy reliance on coal for primary energy consumption. Furthermore, China’s economy is transitioning from high-speed growth to high-quality development. In this crucial period, promoting low-carbon sustainable development and achieving a comprehensive green transformation of the economy and society have emerged as new requirements.

The concept of green development has gradually become deeply ingrained in people’s minds. According to the report of the 20th National Congress of the Communist Party of China, China will accelerate the green transformation of its development model and actively work towards peak carbon dioxide emissions and carbon neutrality. Therefore, given the current landscape of economic and social development, green finance guided by the “dual-carbon” goal holds significant importance for China’s future financial development and infrastructure, making it a cornerstone of China’s national strategies.

## Literature review

The exploration of the relationship between green finance and carbon reduction originated from prior research on the impact of finance on carbon reduction. Currently, there is no consensus on this matter, and there are mainly three perspectives: On the one hand, Gu Hongmei and He Bin (2012) argue that financial development can effectively promote carbon reduction through promoting technological progress, improving resource utilization efficiency and other means (Acheampong A O 2019; Ye Chusheng & Ye Qin 2019). On the other hand, the research conducted by Haseeb A et al. (2018) found that financial development will promote economic development and increase carbon emissions, thus inhibiting carbon reduction (Nasir M A et al. 2019; Zhang Lihua et al. 2017). Based on these studies, Zhong Yi and Sun Kele (2021) suggest that there is a U-shaped relationship between financial development and carbon reduction. Financial development will inhibit carbon reduction in the early stage but will promote carbon reduction after crossing a certain threshold (Yan Chengliang et al. 2016). Green finance, as a further extension of financial concept, has attracted more and more attention from scholars regarding its impact on carbon emissions reduction with the development of green finance.

Currently, green finance in China is still in a stage of exploration and experimentation. In terms of quantitative research on green finance, many scholars have only conducted research on individual indicators such as green loans and green bonds (Li Yunyan et al. 2023; Xiao Liming & Li Xiuqing 2020), and the comprehensive evaluation of green finance is mostly focused on the national or provincial level (Li Chenggang 2023; Su Jing 2022). When it comes to the relationship between the development of green finance and carbon reduction, most scholars only focus on the impact of financial development on carbon reduction from relatively narrow perspectives such as green loans. In addition, when measuring carbon emissions, many scholars tend to use single indicators such as carbon emission intensity (Yu Xulan & Zhou Ying 2023; Zhang Ke et al. 2023). Few scholars have used comprehensive indicators, such as the total factor carbon emission efficiency, to study the impact of green finance on carbon reduction and their non-linear relationship. In addition, in terms of empirical analysis, most scholars prefer to use panel data models to analyze the relationship between green finance and carbon reduction (Fan Decheng & Zhang Xiufan 2022; Liu Feng et al. 2022). However, a limited number of researchers have chosen to use spatial econometric models to analyze the spatial effects between them. Therefore, considering the current development context, this paper will primarily analyze and discuss the following aspects: the spatial distribution pattern of the development status of green finance and carbon reduction in various provinces and cities in China, the spatial correlation between the two, the impact of green finance on total factor carbon emission efficiency, and the spatial effects resulting from it.

## Theoretical analysis and research hypotheses

### Analysis of the impact of green finance on the efficiency of total factor carbon emission reduction

Green finance is a financial tool for regulating the environment in economic and social development by directing capital towards green industries to achieve environmental constraints. Previous research by Li Shanshan and Ma Yanqin (2019) has shown that environmental regulations have both positive and negative impacts on carbon emissions. As an important financial instrument, green finance can regulate the environment and is also an environmental regulation measure. Therefore, green finance has both positive and negative impacts on the efficiency of total factor carbon emission reduction. On the one hand, green finance serves green environmental projects with high investment costs and long payback periods, making it difficult to produce significant effects on carbon emission reduction in the short term. At the same time, as investment increases and output decreases, coupled with the high cost of green transformation, enterprises may actually increase their consumption of fossil energy to avoid these costs, leading to an increase in carbon emissions (Dong Mei & Li Cunfang 2020; Wang Xinkang et al. 2018). On the other hand, in the long run, green finance will force enterprises to engage in green production activities through technological innovation and other means, placing more emphasis on green development in order to obtain more funding support. When the benefits of technological innovation outweigh R&D costs, innovation compensation effects will be generated. Market competition will also make enterprises more actively adjust their industrial structures to gain competitive advantages, thus promoting the development of the green economy, further enhancing the efficiency of total factor carbon emission reduction across society (Porter M E & Linde C 1995; Yu Zhihan et al. 2021).

In conclusion, this article proposes research hypothesis 1: the impact of green finance on the total factor carbon emission reduction efficiency exhibits a non-linear U-shaped relationship.

### Analysis of spatial effects of the impact of green finance on total factor carbon emission reduction efficiency

Green finance, similar to traditional finance, exhibits spatial agglomeration characteristics. Initially, the development of green finance aimed to provide specialized financial services for green environmental projects. By promoting the growth of local green industries, it can facilitate the dissemination of green technologies and related policies to surrounding areas. As a result, it influences the total factor carbon emission reduction efficiency of these neighboring regions through spatial spillover effects. During the early stages of green finance development, a polarization effect can be observed. This means that the central region attracts production factors from the surrounding areas through green policies, leading to a forefront position in low-carbon transformation. However, during this phase, it may negatively impact the carbon emissions reduction efforts of the surrounding regions. Nevertheless, as factors such as capital and labor accumulate in large quantities, competitiveness within the central area increases. Eventually, when the costs of competition outweigh the profits obtained, the development of green finance in the central area spreads to the surrounding regions, yielding a positive effect on their carbon emissions reduction.

At this point, the core area of green finance will disseminate advanced green technologies to surrounding regions, stimulating technological innovation and promoting carbon emissions reduction. Simultaneously, when the local industrial return rate fails to meet capital requirements, funds will be redirected to green industries in the surrounding areas. Financial institutions will increase support for funds, reducing information asymmetry between fund supply and demand. This will widen financing channels for green environmental enterprises in the surrounding regions, facilitating industrial low-carbon transformation and promoting carbon emissions reduction (Yang Chunxia & Ji Hailun 2022).

In conclusion, this article proposes research hypothesis 2: the impact of green finance on the total factor carbon emission reduction efficiency in China exhibits a spatial spillover effect characterized by a U-shaped relationship.

### Analyzing the mechanism of green finance in influencing total factor carbon emission reduction efficiency in China

The efficiency of total factor carbon emission reduction can be decomposed into two components: technological progress (BPC) and changes in technical efficiency (EC). If green finance has an impact on total factor carbon emission reduction efficiency, then technological progress and technical efficiency may serve as intermediate channels through which it influences such efficiency.

Technological progress (BPC) in the economic context refers to the overall advancement of the production frontier, representing the most advanced production technology. This means achieving higher output levels using the same input combination or obtaining the same outputs with fewer inputs. Any economy that pushes its production frontier outward is considered to have achieved technological progress, which can contribute to carbon emission reduction (Li Ping, 2016). Green finance plays a crucial role in promoting technological progress by providing continuous funding for scientific and technological research and development, ensuring sufficient investment in human capital, and improving the management mechanism for allocating research funds and income distribution. These measures enhance the efficiency of carbon emission reduction.

Technical efficiency (EC) refers to maximizing the utilization of existing technology by improving coordination among various resource elements while keeping the technology level unchanged. When enterprises strive to operate at the established optimal technological level within a given technological frontier, they can enhance input-output efficiency and effectively promote carbon emission reduction. Green finance, among other things, can optimize production and operation management by adjusting internal management and organization modes, enhancing employee quality, and accelerating information transmission and processing in all aspects of production and operation. These actions improve the coordination of resource elements, leading to enhanced technological efficiency and further promotion of carbon emission reduction.

However, since green finance primarily focuses on green projects with long recovery cycles, the short-term benefits may not be significant enough. Consequently, the limited inputs during this period might not sufficiently stimulate technological progress and efficiency. Moreover, the increased production burden on enterprises, coupled with the consideration of cost considerations, might lead to reduced investments in technological research and development. Additionally, the market for traditional non-clean technologies still dominates over an extended period, limiting the income benefits from enterprise research and development of green technologies. Consequently, motivation for green technology research and development becomes insufficient, resulting in a negative impact on technological progress and technical efficiency caused by green finance (Dong Zhiqing & Wang Hui, 2019).

Nevertheless, as the effects of green finance policies continue to amplify, enterprises will consciously internalize external costs and increase technological innovation to meet the requirements of green finance. In doing so, they aim to reap the potential benefits of technological innovation. At this stage, the presence of green finance reduces the cost of technological innovation for enterprises, making the pulling effect of technological progress and efficiency increasingly evident, ultimately demonstrating a positive impact (Li Shanshan & Ma Yanqing, 2019; Liu Yi et al., 2020; Guan Hailing & Wu Zhenni, 2020).

Based on the aforementioned analysis, this study proposes research hypothesis 3: green finance exerts a U-shaped impact on the efficiency of total factor carbon emission reduction through technological progress (BPC) and technological efficiency (EC).

## Research design

### Indicator selection and measurement

#### Construction of green finance evaluation indicator system

Based on the understanding of the connotation of green finance and the review of relevant literature, a green finance evaluation indicator system is constructed for China. The indicator system includes five secondary indicators: green credit, green securities, green insurance, green investment, and carbon finance, as well as corresponding tertiary indicators. The specific indicator system is shown in Table 1.

**Table 1:** Green finance evaluation index system

Primary indicator	Secondary indicator	Tertiary indicator	Indicator definition	Indicator attributes
Green finance	Green loans	Level of development of green loans	Energy-saving and environmental protection loans/GRP	Positive
		The proportion of interest expenses in high-energy-consuming industries	Interest on high-energy-consuming industrial industries/industrial interest	Negative
	Green securities	The proportion of market value for environmental protection enterprises	Market value of environmental protection companies/the market value of A-shares	Positive
	Green insurance	Scale ratio of agricultural insurance	Agricultural insurance expenditure/agricultural insurance revenue	Positive
	Green investment	Investment-to-pollution-reduction ratio	investment in industrial pollution control/GRP	Positive
		Investment-to-energy-saving and environmental protection ratio	Investment in energy-saving and environmental protection/fiscal expenditure	Positive
	Carbon finance	Carbon emission intensity	Carbon emissions/GRP	Negative

This article uses a combined method of subjective and objective weighting to determine the weight of each indicator in green finance. The subjective weight refers to the weight used by Li Xiaoxi and Xia Guang (2014) in “China Green Finance Report,” combined with the actual research of this article and related research experience, the subjective weight is determined as shown in Table 2. The objective weight is calculated using the entropy weighting method, and finally, the two weights are weighted and averaged to obtain the final weight (Table 3), that is: Comprehensive Weight = 0.5 * Subjective Weight + 0.5 * Objective Weight.

**Table 2:** Subjective weighting table

	Green credit development level	Proportion of interest payments in high-energy-consumption industries	Proportion of market value of environmental protection enterprises	Proportion of agricultural insurance scale	Pollution control investment ratio	Proportion of energy conservation and environmental protection expenditure	Carbon emission intensity
Subjective weighting	25%	25%	25%	10%	2.5%	2.5%	10%

**Table 3:** Composite weight table

Secondary indicator	Tertiary indicator	Indicator definition	Composite weight
Green finance	Green loans	Level of development of green loans	20.5%
		The proportion of interest expenses in high-energy-consuming industries	15.1%
	Green securities	The proportion of market value for environmental protection enterprises	34.3%
	Green insurance	Agricultural insurance income/agricultural insurance expenditure	8.7%
	Green investment	Investment-to-pollution-reduction ratio	10.4%
		Investment-to-energy-saving and environmental protection ratio	4.9%
	Carbon finance	Carbon emission intensity	6.3%

### Data source

Considering the availability of data and the current status of green finance development in China, this article identifies 30 provinces and cities in China (excluding Tibet, Hong Kong, Macao, and Taiwan) as the research scope. The official implementation of green credit in China began with the release of the “Green Credit Guidelines” in 2008, which marked the beginning of the growth period of green finance development. On the one hand, considering the temporal nature of policies, and on the other hand, taking into account the decline in the resumption rate during the period from 2021 to 2022 caused by the COVID-19 virus compared to 2020, this study excluded the data from these 2 years to prevent highly deviated results. Instead, the sample used for analysis consisted of data from 2010 to 2020.

The data sources for each indicator are as follows:

1. Green loans: Due to the difficulty in obtaining provincial- and city-level green credit data in China, this article refers to the method used by Xu Sheng et al. (2018) to measure the development of green credit in China by using the amount of energy-saving and environmental protection loans as a representative. The data is mainly obtained from the *GuotaiAn database*. However, because it is national-level data, it is difficult to obtain energy-saving and environmental protection loan balance data at the provincial and city levels. Therefore, this article also borrows the idea of calculating the size of private finance by Li Jian and Wei Ping (2015). By assuming that the proportion of energy-saving and environmental protection loan size in each region to the national energy-saving and environmental protection loan size is equal to the proportion of financial institutions’ loans in each region to the national financial institutions’ loans, the energy-saving and environmental protection loan balance of each region is calculated. The data mainly comes from the “China Financial Statistical Yearbook.” The data on interest expenses of high-energy-consuming industries used in the study mainly come from the statistical yearbooks of various provinces and cities and the “China Industrial Statistical Yearbook.” The missing data for 2018 were collected by consulting the “Fourth Economic Census Yearbook.”
2. Green securities: The market value of environmental protection enterprises and the total A-share market value of each province come from the *WIND database*.
3. Green insurance: The data on agricultural insurance expenditures and income come from the “China Insurance Yearbook.”
4. Green investment: The data on the pollution control ratio of industrial pollution and the expenditure on energy-saving and environmental protection come from the “China Statistical Yearbook” and the “China Environmental Statistical Yearbook.”
5. Carbon finance: The data on fossil fuel consumption involved in the indicators come from the statistical yearbooks of various provinces and cities and the “China Energy Statistical Yearbook.” The carbon emissions are calculated by multiplying the energy consumption by the corresponding conversion factor. For missing or unavailable data in the sample, this article uses interpolation and trend extrapolation methods to fill in the missing values.

### Measuring the efficiency of whole-system carbon emissions reduction

After inputs such as energy and labor are invested in production activities, not only economic output will be generated, but also unexpected outputs such as carbon emissions. Kaoru Tone (2001) believes that the traditional DEA model can only measure the efficiency value of decision-making units, which has certain limitations. Therefore, he innovatively proposed the SBM model for research purposes. With increasing attention to carbon emissions, Chunhong et al. (2011), F. Wu et al. (2012), and other scholars believe that the carbon emissions generated by economic output are unavoidable environmental externalities. Therefore, carbon emissions should be included as an unexpected output in the efficiency evaluation model. Thus, the SBM model with unexpected outputs was established. This paper uses the SBM model based on unexpected outputs combined with the GML index to measure the total factor carbon emission reduction efficiency of 30 provinces and cities in China from 2010 to 2020. Relevant input-output variables need to be introduced in the process of measuring the total factor carbon emission reduction efficiency (Table 4).

**Table 4:** Input-output variables table

Variable types	Variable	Specific explanation
Input variable	Labor input	End-of-year employment figures
	Capital investment	Capital stock
	Energy input	Total energy consumption
Expected output variable	GRP	GRP
Unexpected output variables	Carbon emissions	Carbon emissions

The specific explanations for the selected indicators are as follows:

1. Labor input. The end-of-year employment figures were selected to characterize labor input in this article.
2. Capital investment. The capital stock is used in this article to reflect capital input, and the perpetual inventory method is adopted to calculate it, drawing on the processing method of Shan Haojie (2008).
3. Energy input. The total energy consumption is selected to characterize energy input in this paper.
4. Expected output. Taking 2009 as the base year for equalization, the constant price of gross domestic product in each province and city is selected as the expected output.
5. Unexpected output. Carbon emissions are used to measure non-expected output in this paper (Matthew Wegener & Gholam R. 2019; Ma Dalai et al. 2015). The calculation method is to multiply the consumption of eight fossil energy sources by their corresponding conversion coefficients and carbon emission factors, and then add them up.

All data comes from the *National Bureau of Statistics website, various provincial and municipal statistical yearbooks*, the “China Population and Employment Statistics Yearbook” and the “China Energy Statistics Yearbook.”

### Other control variables

This article introduces the following control variables for analysis:

1. Research and Development (R&D) investment. The proportion of government R&D investment to total fiscal expenditure is used in this article to reflect R&D investment.
2. Comprehensive energy consumption. The proportion of energy consumption to regional Gross Domestic Product (GDP) is used in this article to represent the level of comprehensive energy consumption.
3. Human capital. The number of university students per 10,000 people is used in this article to reflect China’s human capital.
4. Infrastructure construction. This article uses the measurement of per capita transportation road area.
5. Government regulation. This article uses the proportion of government fiscal expenditure to regional GDP to represent government regulation.

The data comes from the *website of the National Bureau of Statistics* and the *statistical yearbooks of various provinces and cities*.

### Descriptive statistical analysis of variables (Table 5)

From the table above, it can be seen that there is a large gap in the green finance and total factor carbon emission reduction efficiency of 30 provinces and cities in China from 2010 to 2020. The largest green finance efficiency is 0.537, while the smallest is only 0.100. The largest total factor carbon emission reduction efficiency is 3.246, while the smallest is only 0.843. There is not much difference among the provinces and cities in terms of research investment and human capital, but there is a large gap in the maximum and minimum values in terms of comprehensive energy consumption, infrastructure construction, and government regulation. This indicates that the 30 provinces and cities in China are not balanced enough in terms of energy consumption, infrastructure construction, and government financial investment.

**Table 5:** Descriptive statistics of variables

Variable name	Symbol	Min	Max	Average value	Standard deviation
Overall carbon efficiency of all elements	CMP	0.843	3.246	1.404	0.410
Green finance	Gfinance	0.100	0.537	0.270	0.078
Green finance^2^	AG	0.010	0.288	0.079	0.047
Technological investment	lntech	-5.550	−2.695	−4.087	0.628
Comprehensive energy consumption	power	0.255	2.279	0.836	0.450
Human capital	lnhu	4.381	6.023	5.248	0.269
Infrastructure construction	road	4.040	26.780	15.626	4.798
Government regulation	gov	0.106	0.643	0.247	0.103

### Model setting

This article examines the impact of green finance on total factor carbon efficiency. Green finance is essentially a financial tool used for ecological and environmental protection, as well as a measure of environmental regulation. At present, there is no consensus on the impact of environmental regulation on carbon reduction. This paper uses total factor carbon efficiency as a proxy variable for China’s carbon reduction, and introduces the quadratic term of green finance to study the non-linear relationship between green finance and total factor carbon efficiency. The model also focuses on the spatial spillover effect of green finance on total factor carbon efficiency. The constructed model is as follows:

1 $${CMP}_{it}={\upalpha}_i+\uprho W\ast {CMP}_{it}+{\upbeta}_1\ast Gfinance+{\upbeta}_2\ast AG+{\upbeta}_{control}{X}_{control}+{\updelta}_1W\ast {Gfinance}_{it}+{\updelta}_2W\ast {AG}_{it}+{\updelta}_3W\ast {X}_{control}+{\upgamma}_t+{\upmu}_{it}$$

2 $${EC}_{it}={\upalpha}_i+\uprho W\ast {CMP}_{it}+{\upbeta}_1\ast Gfinance+{\upbeta}_2\ast AG+{\upbeta}_{control}{X}_{control}+{\updelta}_1W\ast {Gfinance}_{it}+{\updelta}_2W\ast {AG}_{it}+{\updelta}_3W\ast {X}_{control}+{\upgamma}_t+{\upmu}_{it}$$

3 $${BPC}_{it}={\upalpha}_i+\uprho W\ast {CMP}_{it}+{\upbeta}_1\ast Gfinance+{\upbeta}_2\ast AG+{\upbeta}_{control}{X}_{control}+{\updelta}_1W\ast {Gfinance}_{it}+{\updelta}_2W\ast {AG}_{it}+{\updelta}_3W\ast {X}_{control}+{\upgamma}_t+{\upmu}_{it}$$

Among them, μ_*it*_ represents the random error term. ρ is the spatial lag coefficient of the explanatory variable of total factor carbon emission reduction efficiency, indicating the impact of adjacent regions’ total factor carbon emission reduction efficiency on the local total factor carbon emission reduction efficiency. β_1_and β_2_ are the spatial lag coefficients of green finance and its quadratic term, representing the impact of adjacent regions’ green finance on total factor carbon emission reduction efficiency. *W* ∗ *Gfinance*_*it*_ and *W* ∗ *AG*_*it*_ are the spatial lag terms obtained by multiplying the green finance and its quadratic term of 30 provinces and cities in China by the spatial weight matrix, while *W* ∗ *X*_*control*_ represents the corresponding spatial lag term of the control variable. δ_1_ and δ_2_ are the spatial lag coefficients of green finance and its quadratic term, and δ_3_ is the spatial lag coefficient of the control variable. α_*i*_, ρ, β_1_, β_2_, β_*control*_, δ_1_, δ_2_, and δ_3_ are the coefficients to be estimated.

## Empirical results analysis and discussion

### Spatial correlation analysis

#### Global spatial correlation analysis

This article uses global Moran’s *I* to test the spatial correlation between green finance and total factor carbon emission efficiency, and uses a geographic distance spatial weight matrix to calculate Moran’s *I* index. The results are shown in Table 6.

**Table 6:** Global spatial Moran’s index of green finance and carbon emission reduction efficiency with all factors

Year	Green finance index		Overall carbon emission reduction efficiency	
	Moran’s *I*	*P* value	Moran’s *I*	*P* value
2010	−0.047	0.429	0.120	0.048
2011	−0.015	0.388	0.134	0.036
2012	0.006	0.300	0.101	0.074
2013	0.032	0.211	0.122	0.048
2014	0.024	0.253	0.120	0.050
2015	0.090	0.089	0.108	0.068
2016	0.155	0.024	0.104	0.074
2017	0.196	0.008	0.107	0.070
2018	0.113	0.060	0.115	0.059
2019	0.158	0.021	0.122	0.050
2020	0.135	0.039	0.110	0.064

The values reported above are the values of statistics, and ***, **, and * respectively indicate statistical significance at a significance level of 1%, 5%, and 10%

From Table 6, it can be seen that China’s green finance Moran’s Index showed a mainly upward trend between 2010 and 2020, and the significance also increased. Especially since 2015, Moran’s Index of green finance has been significantly positive at a significance level of 10%, indicating that the spatial correlation of green finance among the 30 provinces and cities in China is positive, and there is a significant spatial clustering phenomenon among different regions. From 2010 to 2020, Moran’s Index of the total factor carbon emission reduction efficiency of the 30 provinces and cities in China was significantly positive at a significance level of 10%, indicating that there is a significant positive spatial correlation in the total factor carbon emission reduction efficiency of China, and the total factor carbon reduction efficiency of each region shows significant positive spatial clustering.

#### Local spatial correlation

Analyze the local spatial correlation of green finance and total factor carbon emission reduction efficiency by drawing Moran’s *I* scatter plots. Due to space limitations, this article only shows the spatial aggregation table of green finance and total factor carbon emission reduction efficiency based on Moran’s *I* scatter plots drawn for each province and city in China in 2016 and 2020. The results are shown in Table 7.

**Table 7:** Spatial agglomeration tables of green finance and total factor carbon emission reduction efficiency for each province and city in 2016 and 2020

	Cluster mode	2016	2020
Green finance	H-H	Beijing, Tianjin, Shanghai, Jiangsu, Anhui, Fujian, Jiangxi, Hubei	Beijing, Tianjin, Jilin, Shanghai, Jiangsu, Anhui, Fujian
	L-H	Hebei, Inner Mongolia, Liaoning, Fujian, Shandong, Hubei, Hunan, Sichuan	Hebei, Inner Mongolia, Liaoning, Fujian, Shandong, Hubei, Hunan, Sichuan
	L-L	Henan, Guangxi, Hainan, Guizhou, Yunnan, Shaanxi, Qinghai, Ningxia, Xinjiang	Henan, Guangxi, Hainan, Guizhou, Yunnan, Shaanxi, Qinghai, Ningxia, Xinjiang
	H-L	Shanxi, Heilongjiang, Jiangxi, Guangdong, Chongqing, Gansu	Shanxi, Heilongjiang, Jiangxi, Guangdong, Chongqing, Gansu
Full-factor carbon emission reduction efficiency	H-H	Shanghai, Jiangsu, Zhejiang, Fujian, Shandong, Henan, Hubei, Hunan, Chongqing	Beijing, Tianjin, Shanghai, Jiangsu, Zhejiang, Fujian, Shandong, Henan, Hubei, Hunan, Chongqing
	L-H	Beijing, Shanxi, Liaoning, Heilongjiang, Anhui, Jiangxi, Hainan, Guizhou	Shanxi, Liaoning, Anhui, Jiangxi
	L-L	Hebei, Inner Mongolia, Guangxi, Yunnan, Shaanxi, Gansu, Qinghai, Ningxia, Xinjiang	Inner Mongolia, Heilongjiang, Hainan, Guizhou, Shaanxi, Gansu, Qinghai, Ningxia, Xinjiang
	H-L	Tianjin, Jilin, Guangdong, Sichuan	Jilin, Guangdong, Sichuan

From the table, it can be seen that there are two clusters of green finance and total factor carbon emission reduction efficiency in China: one is the H-H cluster area mainly formed by eastern regions, where green finance and total factor carbon emission reduction efficiency are relatively high; the other is the L-L cluster area mainly formed by central and western regions, where the level of green finance development and total factor carbon emission reduction efficiency are relatively low. Therefore, it can be concluded that the clusters with high (low) levels of green finance are also the clusters with high (low) total factor carbon emission reduction efficiency.

### Spatial metric results analysis

#### Analysis of the model estimation results

Before conducting regression estimation, this article needs to determine the spatial econometric model to be used by testing, and use the LM test to determine the existence of spatial lag model and spatial error model. From the results of the test in the table, it can be seen that the LM test, robust LM test, LR test, Wald test, and Hausman test all passed the significance test at the 1% level (Table 8), indicating that the fixed-effects spatial Durbin model should be selected.

**Table 8:** LM, LR, Wald, Hausman test results

Verification	Statistical metric	*P* value
LMerr	60.008	0.000
LMlag	38.409	0.000
R-LMerr	35.176	0.000
R-LMlag	13.578	0.000
LR(SDM&SLM)	70.70	0.000
LR(SDM&SEM)	105.65	0.000
Wald(SLM)	18.38	0.000
Wald(SEM)	34.89	0.000
Hausman	52.43	0.000

1. Green finance has a non-linear U-shaped effect on the overall factor carbon emission reduction efficiency.

From Table 9, it can be seen that the coefficient of green finance is significantly negative at the 1% level of significance, but its quadratic term is significantly positive at the 1% level of significance. This indicates that the impact of green finance on the overall factor carbon emission reduction efficiency shows a non-linear U-shaped relationship. Therefore, hypotheses 1 and 2 are validated.

**Table 9:** Results of the Space-Doberman model test

Variable	Coefficient	Standard error	*P* value
Gfinance	−7.520***	1.112	0.000
AG	11.487***	1.737	0.000
lntech	0.133**	0.047	0.005
power	−0.543***	0.124	0.000
lnhu	0.161	0.163	0.323
road	−0.020**	0.007	0.003
gov	0.381	0.504	0.450
W*Gfinance	−6.997***	2.196	0.001
W*AG	14.400***	3.637	0.000
W*lntech	0.623***	0.131	0.000
W*power	−0.968***	0.292	0.001
W*lnhu	−0.007	0.297	0.811
W*road	0.004	0.016	0.809
W*gov	−1.892*	1.076	0.079
Spatial-rho	0.267***	0.079	0.001
sigma2_e	0.021***	0.002	0.000
R-squared	0.777		
Log-likelihood	164.489		

***, **, and * indicate statistical significance at the 1%, 5%, and 10% level, respectively

Analysis is as follows: Firstly, green finance mainly serves ecological protection, and mostly supports green environmental protection projects with larger initial input of factors but with unclear short-term benefits. At this time, the investment exceeds the output, leading to a decrease in the all-factor productivity of green production. Secondly, green environmental protection projects require enterprises to increase their equipment purchase and related technology innovation efforts in pollution control and emission reduction to meet project requirements. If the support for green financing is small, it will lead to insufficient investment and additional costs, thereby increasing the burden on enterprises. This further affects the improvement of the all-factor carbon emission reduction efficiency. Green finance policies will also increase the financing cost of pollution and energy-consuming projects for enterprises. Enterprises can only bear the innovation costs and investment risks alone, resulting in crowding-out effects on enterprise research and development activities, reducing productivity, increasing unexpected outputs, and suppressing all-factor carbon emission reduction efficiency. At this time, the regulatory role played by green finance continues to increase, and the crowding-out effect generated will continue to intensify, weakening enterprise innovation levels. When crossing the turning point of the U-shaped curve, green finance can provide more adequate financial support for enterprises, reduce costs, share risks, greatly improve the innovation enthusiasm of enterprises, and pay more attention to improving production processes and improving technological levels. Enterprises that focus on the development of green finance can better meet the needs of the green development of the economy and society by adopting environmentally friendly technologies and developing relevant products at an early stage, thus occupying competitive advantages in the market and offsetting the impact of enterprises paying more costs to meet higher environmental requirements. At the same time, green finance will raise the financing threshold for high-energy-consuming and high-polluting enterprises, forcing them to transform and upgrade their industrial structure, continuously improve resource utilization efficiency, promote cleaner and greener production, reduce unexpected outputs, and thus improve the all-factor carbon emission reduction efficiency. In summary, green finance may have a suppressive effect on the all-factor carbon emission reduction efficiency in the early stage of development.

However, with the development of the green financial system becoming more perfect and mature, it can provide more comprehensive financial services for green projects and have greater support, thus significantly improving the all-factor carbon emission reduction efficiency.

It can be calculated that the inflection point of the U-shaped curve is 0.340. Comparing with the development level of green finance in various provinces and cities in China, it is found that the green finance in Beijing-Tianjin area, eastern coastal areas, and some central provinces and cities such as Heilongjiang, Anhui, Chongqing, and Jiangxi have exceeded the inflection point value. This can significantly improve the local total factor carbon emission reduction efficiency, while the green finance in other regions plays a restraining role in the local total factor carbon emission reduction efficiency. Therefore, it can be concluded that there are regional differences in the impact of green finance on the total factor carbon emission reduction efficiency in China at present.

(2) Spatial lag effect analysis of total factor carbon emission reduction efficiency.

According to Table 9, the spatial auto-correlation coefficient (rho) of the total factor carbon emission reduction efficiency is 0.267 and has passed the significance test at the 1% level. This indicates that a 1% increase in the total factor carbon emission reduction efficiency in surrounding areas will lead to a 0.267% increase in the local total factor carbon emission reduction efficiency. Therefore, there is a significant spatial interaction effect among the total factor carbon emission reduction efficiencies of different regions. Regions with high total factor carbon emission reduction efficiency tend to cluster together and can drive the improvement of the total factor carbon emission reduction efficiency in neighboring areas through radiation effects. The probability of policy imitation between adjacent regions is higher. When one region implements carbon emission reduction development policies, it will encourage neighboring regions to take action, thereby forming a virtuous competition and promoting coordinated development of regional green environmental protection.

(3) Spatial spillover effects analysis of green finance on total factor carbon emission efficiency.

As shown in Table 9, the spatial effect coefficients of green finance and its quadratic term are −6.997 and 14.400, respectively, both passing a significance test at the 1% level, which indicates that the impact of green finance on the total factor carbon emission efficiency of surrounding areas exhibits a U-shaped pattern. In the early stages of green finance development, the green finance of surrounding areas had a certain inhibitory effect on the total factor carbon emission efficiency of the local area. However, when the development level of green finance was relatively high, it had a positive promoting effect on the total factor carbon emission efficiency of the local area.

Therefore, it can be seen that there is a spatial spillover effect of green finance on China’s total factor carbon emission efficiency, which shows a U-shaped relationship.

From the regression results of the explanatory variable spatial lag, it can be obtained that the U-shaped inflection point value of adjacent green finance on the total factor carbon emission efficiency of the local area is 0.243. Comparing the calculated results of green finance in various provinces and cities, we found that most regions of China have exceeded this inflection point value, indicating that green finance in most provinces can promote the improvement of total factor carbon emission efficiency of surrounding provinces.

Based on the estimation results of the spatial Durbin model, the spatial effects were decomposed and analyzed into direct and indirect effects, and the test results are shown in Table 10. From the table, it can be seen that the improvement in green finance development in the region shows a U-shaped relationship with the improvement in the total factor carbon emission reduction efficiency in the local area, and the spillover effect on the improvement of the total factor carbon emission reduction efficiency in the surrounding areas also exhibits a U-shaped pattern. As an environmental regulation measure, green finance, according to the “pollution haven hypothesis,” when a country’s environmental regulation policy becomes stricter, it will cause the transfer of polluting industries to countries with less stringent environmental regulations to avoid cost constraints. Therefore, it can be inferred that when the level of green finance in a certain region increases, polluting industries will migrate to adjacent regions with lower levels of green finance, thus reducing the total factor carbon emission reduction efficiency in adjacent areas. However, in the long run, after the regions that undertake polluting industries improve their economic level, they will also increase their awareness of green development, and policy imitation between adjacent regions is stronger. Regions with high levels of green finance will prompt adjacent regions with lower development levels to imitate green finance policies, thereby promoting the improvement of carbon emission reduction efficiency. In conclusion, the green finance of a region can have an impact on both the local area and its surrounding areas. Therefore, to improve the total factor carbon emission reduction efficiency in the local area, the level of green finance in the local area and adjacent areas should be comprehensively considered to promote regional coordinated development.

(4) Analysis of the influence of other control variables.

**Table 10:** Test results for direct and indirect effects

Variable	Direct effect	*P* value	Indirect effect	*P* value	Overall effect	*P* value
Gfinance	−7.921***	0.000	−11.925***	0.000	−19.846***	0.000
AG	12.263***	0.000	23.188***	0.000	35.451***	0.000
lntech	0.170***	0.000	0.862***	0.000	1.0315***	0.000
power	−0.597***	0.000	−1.437***	0.000	−2.034***	0.000
lnhu	0.158	0.302	−0.040	0.911	0.117	0.749
road	−0.020**	0.003	−0.001	0.967	−0.021	0.361
gov	0.312	0.537	−2.365	0.089	−2.053	0.133

***, **, and * indicate statistical significance at the 1%, 5%, and 10% level, respectively

From Table 9, it can be seen that scientific research investment has a significant positive effect on the efficiency of total factor carbon emission reduction, and a larger scientific research investment is more conducive to promoting carbon reduction. Both comprehensive energy consumption and infrastructure construction have a significant negative impact on the efficiency of total factor carbon emission reduction. The impact of human capital and government regulations on the efficiency of total factor carbon emission reduction is positive, but the significance of the results is not strong.

#### Robustness test

In the previous section, a spatial econometric model was established using a geographic distance spatial weight matrix. In order to verify the robustness of the model, an adjacency 0-1 spatial weight matrix is introduced in the following section, and the variables mentioned above are inserted into the model for testing. The test results are shown below.

From Table 11, it can be seen that the coefficients of green finance and its quadratic term, as well as the coefficients corresponding to the lagged term of the carbon emission efficiency of both variables, are significant, and the direction of the signs is consistent with the previous text. From Table 12, it can be seen that the direction of the coefficients of green finance and its quadratic term in all three effects is consistent with the previous text, and all have passed the significance level test of 1%. In summary, the spatial Durbin model constructed in this paper is robust.

**Table 11:** Results of robustness test for spatial Durbin model (1)

Variable	Coefficient	Standard error	*P* value
Gfinance	−7.140***	1.063	0.000
AG	10.759***	1.677	0.000
lntech	0.205***	0.048	0.000
power	−0.511***	0.132	0.000
lnhu	−0.198	0.180	0.270
road	−0.016**	0.007	0.026
gov	0.542	0.513	0.291
W*Gfinance	−4.279**	2.066	0.038
W*AG	10.945**	3.547	0.002
W*lntech	0.173*	0.093	0.063
W*power	−0.639**	0.220	0.004
W*lnhu	0.326	0.263	0.215
W*road	0.028**	0.012	0.026
W*gov	−1.351	0.834	0.105
Spatial-rho	0.244***	0.068	0.000
sigma2_e	0.022***	0.002	0.000
R-squared	0.765		
Log-likelihood	156.855		

***, **, and * indicate statistical significance at the 1%, 5%, and 10% levels, respectively

**Table 12:** Results of robustness test for spatial Durbin model (2)

Variable	Direct effect	*P* value	Indirect effect	*P* value	Overall effect	*P* value
Gfinance	−7.473***	0.000	−7.649***	0.000	−15.122***	0.000
AG	11.520***	0.000	17.282***	0.000	28.802***	0.000
lntech	0.223***	0.000	0.274**	0.005	0.497***	0.000
power	−0.558***	0.000	−0.942***	0.001	−1.500***	0.000
lnhu	−0.185	0.267	0.347	0.225	0.162	0.554
road	−0.014**	0.045	0.031**	0.044	0.017	0.291
gov	0.480	0.345	−1.542	0.125	−1.062	0.275

***, **, and * indicate statistical significance at the 1%, 5%, and 10% levels, respectively

### Analysis of the influence mechanism of green finance on china's total factor carbon emission reduction efficiency

Based on hypothesis 2, this paper utilizes the technical efficiency change index and technical progress index as intermediate variables, alongside the green financial development index and its quadratic term as explanatory variables. The regression models (Model 2 and Model 3) are estimated, incorporating the aforementioned control variables.

The results of the regression analysis are presented in Table 13 and Table 14, verifying hypothesis 3. Analyzing the influence mechanism of green financial development on total factor carbon emission reduction efficiency reveals several key findings. Firstly, from the perspective of green financial development, the coefficients of green finance in both the technical efficiency change index and technical progress index models are significantly negative at the 5% significance level. Additionally, the coefficients of the quadratic term of green finance in both models are significantly positive at the 1% significance level. These findings indicate a non-linear U-shaped relationship between green finance and both the technical efficiency change index and technical progress index.

**Table 13:** Empirical results on impact mechanisms—EC

Variable	Coefficient	Standard error	*P* value
Gfinance	-0.140***	0.0308	0.000
AG	0.0309***	0.0069	0.000
lntech	0.0767**	0.0275	0.009
power	-0.409***	0.0790	0.000
lnhu	0.0771	0.0655	0.249
road	-0.0038	0.0029	0.204
gov	-0.0719	0.186	0.702
cons	1.589***	0.287	0.000

***, **, and * indicate statistical significance at the 1%, 5%, and 10% levels, respectively

**Table 14:** Empirical results on impact mechanisms—BPC

Variable	Coefficient	Standard error	*P* value
Gfinance	-3.620**	1.542	0.026
AG	6.149**	2.367	0.015
lntech	0.0294	0.0577	0.614
power	0.0609	0.201	0.764
lnhu	-0.377 q	0.223	0.102
road	-0.0190*	0.0099	0.064
gov	-1.220*	0.618	0.058
cons	3.971**	1.182	0.002

***, **, and * indicate statistical significance at the 1%, 5%, and 10% levels, respectively

Specifically, during the early stages of green finance development, it exerts a certain inhibitory effect on technological change and improvements in technical efficiency due to insufficient support and limited short-term benefits. Consequently, enterprises often prioritize compliance with environmental regulations through methods such as paying fines for pollution, which dampens their motivation to engage in technological innovation, resulting in a negative impact on carbon emission reduction efficiency.

However, as green finance development reaches a certain threshold, enterprises develop positive expectations regarding green finance policies. They proactively invest in technological innovation to capitalize on potential benefits, leading to an innovation compensation effect and a positive impetus for technological change and improvements in technical efficiency. Moreover, green finance provides crucial financial support and enhances the coordination of various resource elements, further stimulating technological progress and technical efficiency.

Due to the U-shaped relationship between green finance development and both technical efficiency change and technological progress, it follows that green finance development also exhibits a U-shaped impact on total factor carbon emission reduction efficiency.

### Analysis of the influence of other control variables

The input of scientific research has a positive effect on both technical efficiency change and technical change. However, its effect on technical change is not significant, while it is significantly positive at a 5% significance level on technical efficiency change. Therefore, scientific research input mainly affects the total factor carbon emission reduction efficiency by influencing the technical efficiency change.

Comprehensive energy consumption has a significantly negative effect on efficiency change at a 1% significance level, but its effect on technical change is positive and insignificant. This may be because in the short term, an increase in energy inputs can meet the demands of production technology and thus enhance technical change. However, this increase in energy inputs also leads to more non-desired outputs, reducing technical efficiency. By comparing the coefficients of comprehensive energy consumption in the two equations, we can observe that the effect on technical efficiency change is greater than the effect on technical change, resulting in a negative impact of comprehensive energy consumption on the efficiency of total factor carbon reduction.

The effect of human capital on technical efficiency change is negative, while its effect on technical change is positive, but neither effect is significant. This may be attributed to the fact that in the short term, the increased investment in high-quality labor for research work takes longer to manifest its effects. Additionally, the current proportion of highly educated personnel in China is relatively small. As a result, human capital has a disadvantageous impact on technical efficiency changes in specific production scenarios, with its negative effect on technical efficiency being greater than its positive effect on technological change. Consequently, human capital has a negative influence on the total factor carbon emission reduction efficiency, contributing to the negative effect of comprehensive energy consumption on total factor carbon emission reduction efficiency.

Infrastructure construction has a significantly negative effect on both technical efficiency change and technical change at a 10% significance level. This might be due to the immaturity of China’s new energy automobile industry, which primarily relies on traditional energy-consuming transportation modes. As a result, it negatively affects both technical efficiency change and technical change, leading to a significant negative impact on the efficiency of all-factor carbon emission reduction.

Government regulation shows a non-significant negative effect on technical efficiency change and a significant negative effect on technical change at a 10% significance level. This exerts a more substantial inhibitory effect on the total factor carbon emission reduction efficiency. The rationale behind this could be that China’s current investment in technological innovation is extensive and, to some extent, promotes technological progress. However, with the simultaneous increase in investment, the importance placed on technical efficiency is not yet sufficiently high.

## Conclusion and recommendations

This article measures and analyzes the green finance and total factor carbon reduction efficiency in various provinces and cities of China over the past decade, and constructs a spatial panel data model to study the impact of green finance on total factor carbon reduction efficiency. The research results show that the current impact of green finance on total factor carbon reduction efficiency in China is not a simple linear relationship, but presents a U-shaped relationship. In the short term, green finance at a low level has inhibitory effects on total factor carbon reduction efficiency, while in the long term, green finance can significantly improve total factor carbon reduction efficiency after reaching a certain level. In addition, there is a positive spatial lag effect on green total factor productivity, and there is also a U-shaped spatial spillover effect of green finance on total factor carbon reduction efficiency. Research investment, human capital, and government regulation have a promoting effect on total factor carbon reduction efficiency, while comprehensive energy consumption and infrastructure construction have an inhibitory effect on the development of total factor carbon reduction efficiency. Based on this, the following measures are recommended:

1. Improve the green finance system and promote diversified innovation and development of products.

Regions should strengthen the quality of green financial services, improve related infrastructure, and promote the diversified development of green financial products. In order to plan the long-term development of green finance, improve green financial services and expand the scope of green financial services. Government departments should improve the green financial innovation mechanism, encourage financial institutions to carry out innovative activities, and broaden financing channels for enterprises engaged in green environmental protection projects. At the same time, enhance society’s understanding of green finance and increase the influence and credibility of green finance. Establish an interest-free system for green loans, as well as a risk compensation mechanism to attract private financial institutions to participate in green finance construction.

(2) Improve the system for sharing information on green finance and promote coordinated regional development of green finance.

Firstly, it is necessary to improve the system for sharing information on green finance. This involves improving the disclosure system for enterprise information, encouraging enterprises to participate in the construction of information sharing platforms, and increasing the disclosure of financial and environmental information. Information databases should be established for financial institutions, enterprises, and other entities involved in green finance, with a view to improving the mechanisms for sharing information between financial institutions and enterprises. Additionally, the role of regional development coordination should be fully realized by actively building regional cooperation platforms. This can be achieved through various means such as holding green finance seminars and project training sessions, promoting exchanges among green finance practitioners at the regional level, establishing regional green finance pilot zones, creating green finance development alliances, and promoting information exchange and technology sharing.

Drawing on the experience gained from mature regions in green finance development, it is necessary to strengthen cooperation between regions and promote the coordinated development of green finance across regions.

(3) Promote technological innovation and enhance the efficiency of total factor carbon reduction.

The development of green finance will influence technological innovation and thus affect the efficiency of total factor carbon reduction. Financial institutions can promote technological reform and innovation, increase funding support for green product research and development, and encourage enterprises to actively engage in technological innovation and upgrading. At the same time, the government can implement green finance–oriented policies, increase fiscal support for resource-saving and environmentally friendly industries, direct funds towards the green industry, increase taxation on high-energy-consuming and highly polluting enterprises, and compel them to actively engage in technological innovation and accelerate transformation and upgrading, thereby reducing carbon emissions and other pollutants and improving the efficiency of total factor carbon reduction.

(4) Follow regional heterogeneity and maximize emission reduction according to local conditions.

Currently, there is significant potential to improve the level of green finance in central and western China. To achieve the “dual-carbon” goal, it is crucial to establish a green financial investment and financing system that aligns with regional characteristics. Firstly, it is essential to actively promote pilot initiatives in green finance, increasing government and financial institution support for its development. This can be achieved by guiding greater participation of social capital through measures such as carbon subsidies, carbon tax reductions, and the establishment of green finance pilot zones. Such efforts will help stimulate the efficient allocation of green financial resources in the capital market, providing reliable capital support for the realization of the “double carbon” goal.

Secondly, there is a need to foster innovation in green financial tools and expand the range of green financial products and services. Emphasis should be placed on continuous innovation of green financial tools, the creation of diverse green financial products, and the development of businesses related to green credit, green funds, green bonds, green leasing, and green insurance. By building a comprehensive, multi-level, and long-term green financial support system, multiple dimensions of assistance can be effectively provided to contribute towards achieving the “dual-carbon” goal.

In the eastern part of China, which possesses advantages in terms of capital, human resources, and technology, it is recommended to leverage the momentum of fintech development. Specifically, efforts should be made to accelerate the deep integration of green finance and fintech, enhancing the efficiency of investment and financing processes conducted by financial institutions. This will enable the eastern region to capitalize on fintech advancements, furthering the integration of green finance principles and technologies for more effective investment and financing outcomes.

### Supplementary Information

ESM 1: (XLSX 97 kb)

## Data Availability

Due to challenges encountered in acquiring provincial- and city-level green credit data, this study relies on national-level data from reputable sources such as the *GuotaiAn database*, *Wind database*, *the China Insurance Yearbook*, *the China Statistical Yearbook*, *the China Environmental Statistical Yearbook*, *the China Energy Statistical Yearbook*, and *the China Financial Statistical Yearbook*. The interest expenses associated with high-energy-consuming industries are derived from statistical yearbooks encompassing various provinces and cities, including *the China Industrial Statistical Yearbook*. In situations where data for the year 2018 were absent, supplementary information was obtained from *the Fourth Economic Census Yearbook*. All authors confirm that the data supporting the results of this study can be found in the article, its supplementary materials, and publicly available sources.
